# FDD Channel Estimation Via Covariance Estimation in Wideband Massive MIMO Systems

**DOI:** 10.3390/s20030930

**Published:** 2020-02-10

**Authors:** José P. González-Coma, Pedro Suárez-Casal, Paula M. Castro, Luis Castedo

**Affiliations:** Department of Computer Engineering & CITIC Research Center, University of A Coruña, 15001 Galicia, Spain; pedro.scasal@udc.es (P.S.-C.); paula.castro@udc.es (P.M.C.); luis.castedo@udc.es (L.C.)

**Keywords:** mmWave, massive MIMO, hybrid architectures, FDD, channel estimation, beam squint

## Abstract

A method for channel estimation in wideband massive Multiple-Input Multiple-Output systems using hybrid digital analog architectures is developed. The proposed method is useful for Frequency-Division Duplex at either sub-6 GHz or millimeter wave frequency bands and takes into account the beam squint effect caused by the large bandwidth of the signals. To circumvent the estimation of large channel vectors, the posed algorithm relies on the slow time variation of the channel spatial covariance matrix, thus allowing for the utilization of very short training sequences. This is possibledue to the exploitation of the channel structure. After identifying the channel covariance matrix, the channel is estimated on the basis of the recovered information. To that end, we propose a novel method that relies on estimating the tap delays and the gains as sociated with each path. As a consequence, the proposed channel estimator achieves low computational complexity and significantly reduces the training overhead. Moreover, our numerical simulations show better performance results compared to the minimum mean-squared error solution.

## 1. Introduction

Massive Multiple-Input Multiple-Output (MIMO) and millimeter wave (mmWave) technologies are promising candidates to satisfy the demands of future wideband wireless communication systems. A common feature of these technologies is the deployment of antenna arrays with a large number of elements while keeping the size of the antenna aperture small. This way, advantages such  as  large beamforming gains necessary to compensate the propagation losses at mmWave frequencies, or the ability to support many spatially multiplexed streams, are efficiently achieved [1,2]. In addition, it has been shown that in multi-cell massive MIMO systems the capacity increases without bound as  the number of antennas increases, even under pilot contamination, if the channel covariance matrices of the contaminating users are asymptotically linearly independent [3]. Measurements campaigns have proved that this is usually the case in practice [4].

Deploying massive antenna arrays raises concerns in terms of hardware cost and power consumption, especially at mmWave frequencies. To alleviate these requirements, hybrid architectures with an analog preprocessing network operating in the Radio Frequency (RF) domain have been proposed to reduce the number of complete RF chains [2]. Hybrid approaches imply stringent limitations, such as the reduced spatial dimensionality and the lack of flexibility of the analog part, usually implemented with a Phase Shifter (PS) network. Thus, results obtained for fully digital scenarios are not applicable to the hybrid case in general, and new precoding or combining solutions are needed.

The benefits of large antenna arrays rely on the accuracy of the Channel State Information (CSI). Considering wideband systems with multicarrier modulations, this information should be acquired for the whole frequency band. However, acquiring the CSI is challenging in this  scenario, especially when the system operates in Frequency-Division Duplex (FDD) mode. In this  case, if the channel is sounded in the downlink, the length of the training sequence depends on the number of transmit antennas [5]. Thus, most prior art on channel estimation focused on systems operating on Time-Division Duplex (TDD) mode [6]. Moreover, the aforementioned restrictions related to hybrid architectures are even more challenging on wideband scenarios, since the analog network is frequency-flat [7]. This‘is incompatible with methods independently designing the spatial signature of the training sequences for each subcarrier, such as those employed in [8,9], or fully digital narrowband solutions. Therefore, hybrid approaches usually compensate these limitations with longer training overheads [10]. This problem is even more relevant if large bandwidths are considereddue to  the beam squint effect [11], which causes that the channel statistics change in each subcarrier.

A common approach to reduce the training overhead is to as sume that the channel is sparse [12,13,14,15]. Some authors conducting measurement experiments reported sparsity at mmWave frequencies, with a few channel propagation paths and small angular spreads [16,17]. Nevertheless, measurement campaigns on sub-6 GHz frequencies with local scatterers around the Base Station (BS) show that these channels are not sparse in general [10,18]. As a consequence, dropping the sparsity as sumption enables a wider applicability, and allows us to circumvent the power leakage typically arisingdue to  basis mismatching.

Other authors consider that the channel second order statistics are known in a dvance. Unfortunately, these channel statistics have to be acquired too in practical settings. In case the channel is almost spatially uncorrelated or Rician-distributed, it has been proved that it is a good approximation to estimate only the diagonal elements of the covariance matrix of the channel [19,20]. Under correlated channels with Rayleigh fading, the training sequences are designed according to the main eigenvectors of the downlink channel covariance [21,22]. However, these training sequences cannot be used in hybrid architectures given their hardware constraints. In TDD scenarios, this covariance can be computed from the uplink [23,24] and extrapolated to the downlink by means of channel reciprocity. However, uplink and downlink channels are different in FDD mode, and in that case angular reciprocity is assumed [25,26,27,28,29]. However, the computational complexity of these solutions for fully-digital narrowband scenarios is high, and they require an additional training period in the downlink to obtain the instantaneous channel realizations.

Channel covariance estimation techniques usually rely on the assumption of Toeplitz covariance matrices, a circumstance that occurs in usual antenna arrangements, such as Uniform Linear Array (ULA), and channel as sumptions, such as Rayleigh fading and the one-ring channel model [19,21,27]. This structure can be exploited to design short training sequences based on sparse rulers. Previous works designed these rulers using coprime arrays [30,31], but this strategy cannot attain the shortest possible training lengths for any number of antennas. There are works that consider more efficient ruler designs for small covariance matrices [32,33], but they lack an efficient method to design these training sequences for an arbitrary number of antennas.

Regarding frequency selective channels, solutions have been proposed [15,34,35]. In [34], authors address covariance estimation in TDD mode for hybrid architectures. The proposed algorithm is an application of Orthogonal Matching Pursuit (OMP) and solves the Multiple Measurement Vector (MMV) problem by allowing for a dynamic sensing matrix. In addition, this method is extended to wideband scenarios under the assumption of common subcarrier support, as  done in [8,14,15]. Conversely, authors consider approximately frequency flat second order channel statistics in [35]. These  as sumptions are impractical for scenarios where the signal bandwidth is as large as  several GHz [10] because of the beam squint effect. Recently, refs. [28,29] considered the wideband scenario under beam squint and relied on angle reciprocity and channel sparsity to estimate the downlink channel in FDD. However, this approach shares the same drawbacks as  other works based on similar as sumptions, namely, large computational complexity and additional training in the downlink, as  explained above.

In this work, we propose a downlink channel estimation method for wideband massive MIMO systems over hybrid architectures based on the explicit acquisition of the channel covariance matrix. With the aim of achieving low training overheads, the proposed method employs training sequences based on sparse rulers. The considered setup typically arises in mmWave frequencies, but the proposed framework is general enough to be applicable in sub-6 GHz bands since channel sparsity is not required. Indeed, our approach only as sumes (1) the channel is wide-sense stationary over a period of time, and (2) the channel covariance matrix has a Toeplitz structure. Moreover,  we consider the nonlinear spatial transformation caused by the beam squint effect. In the following, we summarize the contributions of our work:

- We propose a wideband downlink channel covariance estimation method for hybrid FDD massive MIMO systems that can be utilized for mmWave frequencies. The covariance matrices for different subcarriers lie in distinct subspacesdue to  the beam squint effect. The proposed covariance estimation algorithm implements dictionary rotations to overcome this difficulty and exploit the common structure of the wideband covariance matrices.
- Differently from the prior art, and with the aim of reducing the training overhead, Wichmann sparse rulers [36] are used to design the training sequences. Prior work made use of coprime arrays, but this solution cannot attain the lowest training lengths for a given number of transmit  antennas. Other works that relied on sparse rulers did not provide methods to design the training sequences for a number of antennas arbitrarily large.
- We analyze the limitations of the proposed method based on MUltiple SIgnal Classification (MUSIC): (1) The low rank characteristic of the sample covariance matrices limits the applicability of MUSIC-like techniques. The use of sparse rulers make it possible to extend the proposed method using spatial smoothing techniques and allows for even shorter training sequences;  (2) Prior knowledge on the number of channel paths. We show numerically that the number of channel paths can be estimated without significant performance losses and negligible computational cost. (3) The dictionary dependency. We provide a discussion on dictionary sizes, posing that moderated sizes provide enough angular resolution. Furthermore, since sparsity is not a requirement for the proposed method, we show empirically how to avoid the power leakage arising when the Angle of Departures (AoDs) do not lie on the dictionary.
- We propose a low complexity channel estimator, leveraging on the structure of the wideband channels to avoid the individual per-subcarrier procedure. This estimator obtains the delays and gains corresponding to each channel propagation path exploiting underlying the common information for all the subcarriers. Moreover, the estimation is performed using the training sequence employed for the acquisition of the covariance matrices, and it does not require an additional training stage.

### Notation

In this work, scalars are represented by lower case letters, vectors by bold lower case letters, and matrices by bold capital letters. The superscripts ${}^{T}$ and ${}^{H}$ are the transpose and the Hermitian operators, respectively. The operator |·| with a scalar argument represents the absolute value, ∥·∥ with a vector argument represents its norm and ${∥\cdot ∥}_{F}$ with a matrix argument the Frobenius norm. The operator ⌊·⌋ represents the element-wise floor operation. A⊗B is the Kronecker product of matrices A and B, and A∘B the Khatri–Rao product (or the column-wise Kronecker product) of matrices A and B. $\mathrm{diag}\left(\cdot \right)$ is the diagonal matrix with the arguments in its main diagonal, and $\mathrm{blockdiag}\left(\cdot \right)$ constructs a diagonal supermatrix in which the diagonal elements are given by the matrices in the argument, and the off-diagonal elements are zero matrices. 0 and 1 denote the vectors of zeros and ones of the right dimension, respectively. The constant *c* is the speed of light.

## 2. System Model

We consider a hybrid FDD massive MIMO system with *M* transmit antennas and $N_{RF}$ RF chains communicating with several single-antenna users over a multipath channel. To overcome the channel frequency selectivity, we assume an Orthogonal Frequency-Division Multiplexing (OFDM) modulation with $N_{c}$ subcarriers and a cyclic prefix large enough to avoid intercarrier and intersymbol interference. One of the aims of this work is to estimate the downlink channel covariance; hence, as suming that the channel is locally stationary, we will consider several blocks k∈{1,…,K} with the duration of the channel coherence time. At the beginning of each block, the BS transmits a training sequence of length $T_{\mathrm{tr}}$ OFDM symbols to sound both the covariance and the channel. The remaining time is devoted to data transmission. That is, we collect *K* wideband observations of size $T_{\mathrm{tr}}$ during *K* training periods. Since the block duration is limited by the channel coherence time, the training sequence length must satisfy $T_{\mathrm{tr}}\ll M$ due to  the large antenna array deployed at the BS. Indeed, we circumvent the requirement of training sequence lengths larger than the number of paths *L*, i.e., we propose solutions to the scenario $T_{\mathrm{tr}}\leq L$.

Finally, we will as sume that the users feed the observations back to the BS using, e.g., scalar quantization [8,37]. With the received feedback, the BS estimates the channel covariance matrices for all users and frequency subcarriers. This scheme allows for tracking the channel covariance matrices using subsequent received feedbackdue to  the slow variation of the channel statistics. As previously mentioned, these slow changes make channel covariance acquisition simpler than estimating the channel itself. Next, with the aid of the obtained channel covariance matrices, we estimate the wideband channels employing the  *K* aforementioned observations.

### 2.1. Channel Model

We represent the channel response to an arbitrary user using a model similar to that in [34,38]. We as sume a multipath channel model with *N* taps, where the gains for all antennas in the *f*-th subcarrier during the *k*-th coherence block are given by

(1) $$\begin{array}{cc} h_{k}\left(f\right) & =\sum_{n=0}^{N-1}h_{n,k}\left(f\right)e^{-j2\pi fn/N_{c}},\;\;f=0,\ldots ,N_{c}-1, \end{array}$$

where $h_{n,k}\left(f\right)\in C^{M}$ are the channel coefficients of the *n*-th tap of the channel. Each tap, in turn, is decomposed into *L* paths as 

(2) $$h_{n,k}\left(f\right)=\sum_{l=1}^{L}g_{l,k}p_{\mathrm{rc}}\left(nT_{s}-\tau_{l}\right)a\left(\vartheta_{l},f\right),$$

where the $a\left(\vartheta_{l},f\right)\in C^{M}$ are the steering vectors for the AoD of the *l*-th path $\vartheta_{l}$, $g_{l,k}∼N_{C}\left(0,\sigma_{l}^{2}\right)$ and $\tau_{l}$ are the complex channel gain and the relative delay corresponding to the *l*-th path, respectively, and $p_{\mathrm{rc}}\left(t\right)$ is the transmit filter as sociated with the Digital to Analog Converter (DAC) sampled with time interval $T_{s}$. We as sume that the channel gains for different paths are statistically independent, i.e., $E\left[g_{l}g_{l^{\prime }}^{*}\right]=\sigma_{l}^{2}\delta \left(l-l^{\prime }\right)$.

Regarding the steering vectors, we consider a ULA configuration together with the so-called beam squint effect that arises for the typical antenna apertures in massive MIMO and large relative signal bandwidths $B/f_{c}$ [11]. We express this effect as  a rotation of the steering vectors with respect to the central frequency $f_{c}$, i.e.,

(3) $$a\left(\vartheta_{l},f\right)=\Gamma \left(\vartheta_{l},f\right)a\left(\vartheta_{l}\right),$$

where $a\left(\vartheta_{l}\right)\in C^{M}$ is the steering vector for $\vartheta_{l}$ in a  ULA, whose elements are ${\left[a\left(\vartheta_{l}\right)\right]}_{m}=e^{-j\frac{2\pi }{\lambda }d\left(m-1\right)\sin\vartheta_{l}},m=1,\ldots ,M,$ with *d* is the distance between two consecutive array elements, λ is the carrier wavelength, and $\Gamma (\vartheta_{l},f)$ is a diagonal matrix with the beam squint rotation coefficients. Under the common as sumption of inter-antenna distance $d=\frac{c}{2f_{c}}$, the elements of $\Gamma (\vartheta_{l},f)$ read as 

(4) $${\left[\Gamma \left(\vartheta_{l},f\right)\right]}_{m,m}=e^{j\pi \sin\vartheta_{l}\left(m-1\right)\left(1+\frac{\Delta_{f}}{N_{c}}\frac{B}{f_{c}}\right)},\;m=1,\ldots ,M,$$

where $\Delta_{f}\frac{B}{N_{c}}$ is the frequency offset of the *f*-th subcarrier with respect to $f_{c}$, and $\Delta_{f}=f-\frac{N_{c}-1}{2}$ is its relative position to the central one. This frequency-dependent rotations allows for exploiting the common structure of the wideband covariance matrices, since the beam squint effect for the subcarriers is isolated as  a rotation of the frequency-independent vectors $a(\vartheta_{l})$.

Combining Equations (1) and (2), the channel frequency response for the *f*-th subcarrier reads as 

(5) $$\begin{array}{cc} h_{k}\left(f\right)= & \sum_{l=1}^{L}g_{l,k}\beta_{l}\left(f\right)a\left(\vartheta_{l},f\right) \end{array}$$

where *N* is the number of delay taps and $\beta_{l}\left(f\right)=\sum_{n=0}^{N-1}p_{\mathrm{rc}}\left(nT_{s}-\tau_{l}\right)e^{-j2\pi fn/N_{c}}$.

Since the actual AoD of the channel are unknown, the linear combination of steering vectors in (5) can be approximated by means of the steering vectors as sociated with *G* predefined angles $\theta_{1},\ldots ,\theta_{G}$ comprised in a  frequency-dependent dictionary $A\left(f\right)=[a\left(\theta_{1},f\right),\ldots a\left(\theta_{G},f\right)]$, that is,

(6) $$\begin{array}{c} h_{k}\left(f\right)=\sum_{l=1}^{L}g_{l,k}\beta_{l}\left(f\right)a\left(\vartheta_{l},f\right)\approx A\left(f\right)B\left(f\right)g_{k}, \end{array}$$

where $g_{k}\in C^{G}$ is a vector with *L* non-zero entries in the positions corresponding to the AoDs, i.e., $g_{k}∼N_{C}\left(0,G\right)$ with $G=\mathrm{diag}(\sigma_{1}^{2},\ldots ,\sigma_{G}^{2})$, and $B\left(f\right)=\mathrm{diag}(\beta_{1}\left(f\right),\ldots ,\beta_{G}\left(f\right))$. Note that equality holds for (6) if the AoDs lie on the dictionary. According to the assumptions regarding the channel statistics, we obtain $h_{k}\left(f\right)∼N_{C}\left(0,C_{h(f)}\right)$. Moreover, we use the approximation in (6) yielding

(7) $$C_{h(f)}\approx A\left(f\right)B\left(f\right)GB^{H}\left(f\right)A{\left(f\right)}^{H}=A\left(f\right)\tilde{G}\left(f\right)A{\left(f\right)}^{H},$$

with $C_{h(f)}$ being Toeplitz, and $\tilde{G}\left(f\right)=B\left(f\right)GB^{H}\left(f\right)$ the effective gains for the *f*-th subcarrier. In the following, we will consider that the AoDs belong to the dictionary, i.e., we as sume $C_{h(f)}=A\left(f\right)\tilde{G}\left(f\right)A{\left(f\right)}^{H}$. The consequences of this as sumption are discussed in Section 4.4.

### 2.2. Channel Observations

We aim to estimate the channel response using hybrid architectures to reduce the power consumption and implementation costs. This is achieved by using a number of RF chains significantly lower than the number of antennas, i.e., $N_{\mathrm{RF}}\ll M$. As a counterpart, the feasible hybrid precoding vectors are restricted to the subspace imposed by the hardware limitations. Considering hybrid precoding, the signal received by the users during the *k*-th training block in the *f*-th subcarrier is

(8) $$\varphi_{k}\left(f\right)=T\left(f\right)h_{k}\left(f\right)+v_{k}\left(f\right)=X\left(f\right)Ph_{k}\left(f\right)+v_{k}\left(f\right),\;\;\;f=0,\ldots ,N_{c}-1,\;\;k=1,\ldots ,K,$$

where $v_{k}\left(f\right)∼N_{C}\left(0,\sigma_{v(f)}^{2}I\right)$ is the noise, $T\left(f\right)=X\left(f\right)P\in C^{T_{\mathrm{tr}}\times M}$ is a matrix whose rows are the training vectors for each of the $T_{\mathrm{tr}}$ training channel uses, such that $T^{T}\left(f\right)=\left[t_{1}\left(f\right),\ldots ,t_{T_{\mathrm{tr}}}\left(f\right)\right]$, $t_{t}\left(f\right)\in C^{M}$, $X\left(f\right)=\mathrm{diag}(x_{1}\left(f\right),\ldots ,x_{T_{\mathrm{tr}}}\left(f\right))$ contains the transmitted pilot symbols in the *f*-subcarrier satisfying $|x_{t}\left(f\right)|=1$, and $P^{T}=\left[p_{1},\ldots ,p_{T_{\mathrm{tr}}}\right]$ is the hybrid precoder. Therefore, each of these training vectors is the result of the product

(9) $$t_{t}\left(f\right)=p_{t}x_{t}\left(f\right)=P_{A,t}p_{D,t}x_{t}\left(f\right),\;\;\;\;t=1,\ldots ,T_{\mathrm{tr}},$$

where $p_{t}$ is the configuration of the precoders during the *t*-th training period of *k*-th training block, and $P_{A,t}\in C^{M\times N_{\mathrm{RF}}}$ and $p_{D,t}\in C^{N_{\mathrm{RF}}}$ denote the analog and digital precoding matrices. Recall that $P_{A,t}$ is flat in the frequency domain. Hence, it is not possible to design independent training sequences for each subcarrier.

After the transmission of the $T_{\mathrm{tr}}$ vectors and the subsequent feedback stage, the channel observation $\varphi_{k}\left(f\right)$ of the *k*-th training period will be available at the BS. Since the channel and the noise vectors are statistically independent, the covariance of the observations is given by

(10) $$\begin{array}{c} C_{\varphi (f)}=T\left(f\right)C_{h(f)}T{\left(f\right)}^{H}+\sigma_{v(f)}^{2}I. \end{array}$$

By considering the channel covariance matrix model in (7), we rewrite the previous equation as 

(11) $$\begin{array}{c} C_{\varphi (f)}=\Psi \left(f\right)\tilde{G}\left(f\right)\Psi^{H}\left(f\right)+\sigma_{v(f)}^{2}I, \end{array}$$

where we introduce the measurement matrices $\Psi \left(f\right)=[\psi_{1}\left(f\right),\ldots ,\psi_{G}\left(f\right)]=T\left(f\right)A\left(f\right)$. When the *K* samples of the random variable of (8) are available at the BS, $C_{\varphi (f)}$ is estimated. Furthermore, given that both Ψ(f) and $\sigma_{v(f)}$ are known, determining $C_{\varphi (f)}$ is equivalent to estimating $\tilde{G}\left(f\right)$.

## 3. Training Design

This section is devoted to the design of training sequences to estimate the channel covariance, together with bounds on their length to guarantee its correct identification under the assumption of a  Toeplitz structure.

Toeplitz covariance matrices of size M×M have *M* different coefficients and a training sequence of length $T_{\mathrm{tr}}$ allows for defining $(T_{\mathrm{tr}}^{2}+T_{\mathrm{tr}})/2$ equations. This establishes the following trivial lower bound on the training length: $T_{\mathrm{tr}}\geq \sqrt{2M+1/4}-1/2$. A necessary condition to achieve this bound is that the training sequence should allow for measuring the correlation between pairs of antennas for any separation. This has a strong connection with complete sparse rulers, defined mathematically as  an ordered integer sequence of with *T* elements $R=(r_{1},\ldots ,r_{T})$, such that $r_{1}=0$ and any $z\leq r_{T}$ can be written as  $z=r_{i}-r_{j}$ for some $r_{i},r_{j}\in R$. In this case, we speak of a complete sparse ruler of length $r_{T}$, and a perfect ruler is a complete ruler that contains the minimum possible number of elements for a given $r_{T}$. It has been shown that a training sequence built from a perfect sparse ruler is sufficient for determining Toeplitz covariance matrices [32,33]. In that case, the length of the training sequence is equal to the number of elements of the ruler, i.e., $T_{\mathrm{tr}}=T$, and the last element of the ruler is $r_{T}=M-1$.

In the following, we summarize some results found in the literature for sparse rulers. First, there exist bounds on the size of perfect rulers for a given length [39].

**Theorem** **1.** *(see [39]) Denoting l(r) as  the minimum number of marks of a complete sparse ruler of length r, $\lim_{r\to \infty }\frac{l^{2}\left(r\right)}{r}$ exists and*

(12) $$\begin{array}{c} 2.434\leq \lim_{r\to \infty }\frac{l^{2}\left(r\right)}{r}\leq \frac{375}{112}\approx 3.348. \end{array}$$

Hence, it is possible to identify a Toeplitz covariance matrix using sparse rulers with a training length that satisfies $\sqrt{2.434M}\leq T_{\mathrm{tr}}\leq \sqrt{3.348M}$ when *M* approaches infinity.r.

Utilizing the bounds provided by (12), we evaluate the efficiency of the training sequences employed in previous works on AoD estimation [30,31]. Let us start by introducing a trivial example of a complete sparse ruler of length $m^{2}$, for any arbitrary *m*. Using the 2m−1 marks $\left(0,1,2,\ldots ,m-1,2m-1,3m-1,\ldots ,m^{2}-1\right)$ provides a length of $m^{2}-1$ different differences. This complete ruler achieves a value of $\lim_{m\to \infty }\frac{{\left(2m-1\right)}^{2}}{m^{2}}=4$, larger than the bound of (12). A similar result is obtained with the use of coprime arrays [30,31]. This method is based on two co-prime numbers *p* and *q*, i.e., mcd(p,q)=1, and on generating the ruler as  the set {mp:0≤m<q}∪{nq:0≤n<p}. This method achieves a training length of $T_{\mathrm{tr}}=p+q$ and allows for identifying pq different lags. Thus, the limiting function reads as  $\frac{{\left(p+q\right)}^{2}}{pq}$. Setting $p=q=\sqrt{M}$ provides the bound $\lim_{M\to \infty }\frac{{\left(2\sqrt{M}\right)}^{2}}{M}=4$. Hence, coprime arrays cannot improve the asymptotic efficiency of the previous trivial complete ruler.

In the literature, we can find some techniques to build sparse rulers for arbitrary lengths satisfying the bound in (12). The following section summarizes a method to obtain rulers that are close to that bounds with very low computational complexity.

### 3.1. Wichmann Rulers

Wichmann [36] proposed a direct method to obtain sparse rulers of any size that, denoted as  the differences between consecutive terms, have the form

(13) $$\begin{array}{c} W=(\underset{r}{\underset{︸}{1,\ldots ,1}},r+1,\underset{r}{\underset{︸}{2r+1,\ldots ,2r+1}},\underset{s}{\underset{︸}{4r+3,\ldots ,4r+3}},\underset{r+1}{\underset{︸}{2r+2,\ldots ,2r+2}},\underset{r}{\underset{︸}{1,\ldots ,1}}) \end{array}$$

for some positive integers *r* and *s* such that 2r−2≤s≤2r+4. The number of elements of this ruler is l(r,s)=4r+s+3 and its length $n\left(r,s\right)=4r^{2}+8r+3+\left(4r+3\right)s$. Hence, if the elements of W are denoted as  $w_{i},i=1,\ldots ,l\left(r,s\right)$, the sparse ruler is $R=(r_{1},\ldots ,r_{l(r,s)+1})$ with $r_{i}=r_{i-1}+w_{i},i=2,\ldots ,l\left(r,s\right)+1$ and $r_{1}=0$.

With a change of variable $s=s^{\prime }+\left(2r-2\right)$, the length expression changes to

(14) $$\begin{array}{cc} n(r,s^{\prime }) & =12r^{2}+6r-3+\left(4r+3\right)s^{\prime }\\ & =n_{\mathrm{lo}}\left(r\right)+\left(4r+3\right)s^{\prime },\;\;\;s^{\prime }=0,\ldots ,6. \end{array}$$

The parameter *r* roughly adjusts the length to intervals starting at $n_{\mathrm{lo}}\left(r\right)$. Then, for each *r* value, the $s^{\prime }$ parameter finely adjusts length in steps of size 4r+3. This allows for finding parameters *r* and $s^{\prime }$ that generate a sparse ruler whose length is as close as  possible to a given value.

Finally, the ruler can be completed up to the desired length, relying on the following observation: it  is always possible to build a ruler that contains all differences in the interval [a,b] with a set of the form $R^{\prime }=\left(0,1,2,\ldots ,c,b-c\left(c+1\right),\ldots ,b-2c,b-c,b\right)$, with $c=\lfloor \sqrt{b-a}\rfloor$. The cardinality of this set is at most 2c+3. In this case, given a Wichmann ruler $R=(r_{1},\ldots ,r_{l(r,s)+1})$ of length n(r,s), and a set $R^{\prime }$ that contains all differences in the interval [n(r,s),M], a new ruler extended to length *M* can be found by $R\cup R^{\prime }$.

### 3.2. Training Based on Sparse Rulers

A training sequence to estimate a Toeplitz covariance matrix can be built from a sparse ruler. In that case, the length of the ruler must be equal to M−1, the number of correlation coefficients. Given a sparse ruler of length M−1 with elements $\left(r_{1},\ldots ,M-1\right)\in Z^{T_{\mathrm{tr}}}$, a training sequence for a Toeplitz covariance matrix can be designed as  $T^{T}\left(f\right)=\left[e_{r_{1}+1},\ldots ,e_{M}\right]$, where $e_{i}$ is the *M*-length indicator column vector of zeros and a one in the *i*-th element.

**Figure 1:**
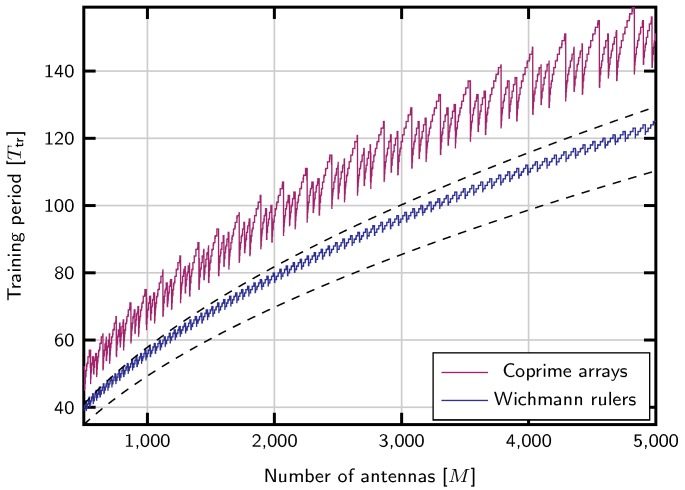
Training length vs. number of antennas with Wichmann rulers. Dashed lines show as ymptotic upper and lower bounds on the minimum training size based on (12).

In the case of Wichmann rulers, it is not possible to obtain them for arbitrary lengths, but instead a length close to a given value. Hence, to obtain a ruler close to length M−1, the *r* parameter can be  found as  the largest such that $n_{\mathrm{lo}}\left(r\right)\leq M-1$, which is $r^{*}=\left\lfloor \frac{\sqrt{12M+33}-3}{12}\right\rfloor$. The other parameter is the largest $s^{\prime }$ such that $n(r^{*},s^{\prime })\leq M-1$, implying that $s^{{}^{\prime }*}=\left\lfloor \frac{M-1-n_{\mathrm{lo}}\left(r^{*}\right)}{4r+3}\right\rfloor$. The length of the ruler can be completed up to M−1 by the method detailed in the previous section. Since the Wichmann rulers always contain entries from 0 to *r*, and the *s* parameter increases length in steps of 4r+3, this completion procedure will add always at most $\left\lceil \frac{4r+3}{r}\right\rceil =5$ marks to the ruler, and the minimum training period will be in the interval $T_{\mathrm{tr}}\in \left[l\left(r^{*},s^{*}\right),l\left(r^{*},s^{*}\right)+5\right]$. Figure 1 shows the training length obtained with this strategy compared to the upper and lower bounds in (12). It also shows the length achieved with the coprime arrays strategy.

In the following, we show that the mild as sumption $N_{\mathrm{RF}}\geq 2$ is enough to obtain the sparse ruler training sequence T(f) according to (9). Let $\phi_{r}$, $r=1,\ldots ,N_{\mathrm{RF}}$ be blueeligible arbitrary phases for the PS_s_ in the analog processing network, and $P_{A,t}^{T}=\left[p_{A,t,1},\ldots ,p_{A,t,M}\right]$, with the $p_{A,t,m}^{T}\in C^{N_{\mathrm{RF}}}$ is the *m*-th row of the analog precoding matrix for the *t*-th channel training use. Since the desired training based on sparse rulers must satisfy $t_{t}\left(f\right)=e_{r_{t}}x_{t}\left(f\right)$, we obtain $P_{A,t}p_{D,t}\left(f\right)=e_{r_{t}}$ as 

(15) $$p_{A,t,m}^{T}=\left\{\begin{array}{cc} {}[e^{j\phi_{1}},e^{j\phi_{1}},e^{j\phi_{2}},\ldots ,e^{j\phi_{N_{\mathrm{RF}}-1}}], & \mathrm{if}\;{\left[e_{r_{t}}\right]}_{m}=1\\ e^{j\phi_{1}},e^{j(\phi_{1}+\pi )},e^{j\phi_{2}},\ldots ,e^{j\phi_{N_{\mathrm{RF}}-1}}], & \mathrm{otherwise}, \end{array}\right.$$

with the digital part as  $p_{D,t}\left(f\right)=\frac{1}{2}e^{-j\phi_{1}}[1,1,\overset{N_{\mathrm{RF}}-2}{\overset{︷}{0,\ldots ,0}}{]}^{T}$. Note that only two elements of $p_{A,t,m}^{T}$ are  employed to achieve $P_{A,t}p_{D,t}\left(f\right)=e_{r_{t}}$. Indeed, $N_{\mathrm{RF}}=2$ is enough to apply (15).

## 4. Covariance Matrix Estimation

In this section, we propose algorithmic solutions to estimate the downlink covariance in wideband massive MIMO systems under the beam squint effect. It is apparent that per-subcarrier (or per-sub-band) covariance estimation is not a practical approach since it does not exploit the structure of the wideband channel. Furthermore, system scalability is compromised when the number of subcarriers is large. The proposed method is based on the estimation of the subspace spanned by the steering vectors corresponding to the *L* AoD of the channel propagation paths. Well-known estimation methods like MUSIC have been employed to estimate a single signal subspace. However, the beam squint effect results in different subspaces for the channel covariance matrices at distinct frequencies. Resorting to the rotation matrices in (4), we extend MUSIC to the considered setup.

### 4.1. Subspace Estimation

To estimate the subspace, let us start by computing the eigendecomposition of the sample covariance matrices ${\hat{C}}_{\varphi (f)}=U\left(f\right)\Lambda \left(f\right)U^{H}\left(f\right)$ for all the frequencies *f*. Given that the covariance converges to the second order moment of the channel for a large number of samples, we have ${\hat{C}}_{\varphi (f)}\to C_{\varphi (f)}$ when K→∞. Next, the eigenvectors corresponding to the *L* largest eigenvalues are discarded to obtain the basis $\bar{U}\left(f\right)$ that spans the noise subspace of the *f* subcarrier. Due  to the beam squint effect, the relationship among dictionary matrices for different frequencies is nonlinear. Therefore, it is not feasible to perform this step jointly for all the subcarriers unless the relative signal bandwidth $B/f_{c}$ is very small. A remarkable feature of this subspace discrimination is its lack of dependency with the SNR, as  long as  the noise is spatially white. Under such as sumption, for the approximation in (11), we get

(16) $$\begin{array}{cc} C_{\varphi (f)} & =\Psi \left(f\right)\tilde{G}\left(f\right)\Psi^{H}\left(f\right)+\sigma_{v(f)}^{2}I_{T_{\mathrm{tr}}}=U\left(f\right)\left(\Lambda \left(f\right)+\sigma_{v(f)}^{2}I_{T_{\mathrm{tr}}}\right)U^{H}\left(f\right). \end{array}$$

Therefore, it is crucial to force this condition by applying a pre-whitening filter, if necessary. According to this observation, the search is performed in the effective subspace, contrarily to OMP-based methods like Covariance OMP (COMP) [34]. Thus, we identify the support $S=\{\theta_{s_{1}},\ldots ,\theta_{s_{L}}\}$, with $s_{i}\in \left\{1,\ldots ,G\right\}\;\forall i$, with the following wideband estimator function

(17) $$J_{i}=\sum_{f=1}^{N_{c}}\frac{1}{∥\psi_{i}^{H}\left(f\right)\bar{U}{\left(f\right)∥}_{2}^{2}},\;\;\;\;i=1,\ldots ,G,$$

where $\psi_{i}\left(f\right)=T\left(f\right)a\left(\theta_{i},f\right)$. Note that this function greatly increases the robustness of the decision, compared to a per-carrier estimator because it exploits the common structure for all the frequencies, given that the vectors $a(\theta_{i},f)$ are generated from a common dictionary as  shown in (3). Finally, the support S is identified as  the *L* largest values of $J_{i}$.

### 4.2. Estimating Gain Variances

Once the angles are determined, we construct the matrices $\Psi_{S}\left(f\right)=T\left(f\right)\left[a\left(\theta_{s_{1}},f\right),\ldots ,a\left(\theta_{s_{L}},f\right)\right]$. Recall that the path delays $\tau_{l}$ of (2) are unknown, as  well as  the matrices B(f) in (7). Therefore, we aim at estimating the per-carrier gain variances $\tilde{G}\left(f\right)$ in (7). In particular, we denote by ${\hat{G}}_{S}\left(f\right)$ the estimated gain variances as sociated with the angles in the support S. Then, it is possible to use the estimator in [40], i.e., ${\hat{G}}_{S}\left(f\right)=\Psi_{S}^{†}\left(f\right)\left({\hat{C}}_{\varphi (f)}-\sigma_{v(f)}^{2}I\right){\left(\Psi_{S}^{†}\left(f\right)\right)}^{H}$. This solution clearly depends on the rank of $\Psi_{S}\left(f\right)$, and cannot be applied in the following situations:

1. $L>T_{\mathrm{tr}}$. This scenario corresponds to short training sequences compared to the number of propagation paths. Under this as sumption, the pseudo-inverse $\Psi_{S}^{†}\left(f\right)$ cannot recover the *L* variance gains.
2. $L\leq T_{\mathrm{tr}}$. In this case, the rank of $\Psi_{S}\left(f\right)$ depends on the choice of the dictionary matrix, A, for a proper training matrix T. Recall that the dictionary contains non-orthogonal vectors when *M* is finite. When the distance between two consecutive angles in the dictionary is small, a situation that arises when *G* is large, this may lead to dictionary matrices such that rank(A)≤min{M,G}, thus making $\Psi_{S}^{H}\left(f\right)\Psi_{S}\left(f\right)$ ill-conditioned.

To circumvent (2), one possibility is to reduce the dictionary size (see Section 4.4). Alternatively, we propose to exploit the diagonal structure of $\tilde{G}\left(f\right)$ and estimate it as

(18) $$\mathrm{vec}\left({\hat{G}}_{S}\left(f\right)\right)={\left(\Psi_{S}^{*}\left(f\right)\circ \Psi_{S}\left(f\right)\right)}^{†}\mathrm{vec}\left({\hat{C}}_{\varphi (f)}-\sigma_{v(f)}^{2}I_{T_{\mathrm{tr}}}\right),$$

with ∘ the Khatri–Rao product (or the column-wise Kronecker product). The product $\Psi_{S}^{*}\left(f\right)\circ \Psi_{S}\left(f\right)$ produces *L*-rank matrices when the columns of  $\Psi_{S}\left(f\right)$ are not co-linear (see Appendix A).

Regarding the channel gain variances, it is again possible to exploit the channel structure to increase the estimation accuracy. Consider the relationship between the values of $\beta_{l}\left(f\right)$ across frequencies, in particular,

(19) $$\begin{array}{c} \beta_{l}\left(f\right)=\sum_{n=0}^{N-1}p_{\mathrm{rc}}\left(nT_{s}-\tau_{l}\right)e^{-j2\pi fn/N_{c}}=\sum_{n=0}^{N-1}p_{\mathrm{rc}}\left(nT_{s}-\tau_{l}\right){\left(e^{-j2\pi \left(-f\right)n/N_{c}}\right)}^{*}=\beta_{l}^{*}\left(-f\right), \end{array}$$

and, denoting $f^{\prime }=-f+N_{c}$, we obtain $\beta_{l}\left(f\right)=\beta_{l}^{*}\left(f^{\prime }\right)$. Recall now that the *L* non-zero diagonal elements of $\tilde{G}\left(f\right)$ are $\beta_{l}\left(f\right)\sigma_{l}\beta_{l}^{*}\left(f\right)={\left|\beta_{l}\left(f\right)\right|}^{2}\sigma_{l},l=1,\ldots ,L$. This implies that $\tilde{G}\left(f\right)=\tilde{G}\left(f^{\prime }\right)$, and  we can increase the robustness of the estimated gain variances in (18) with

(20) $$\begin{array}{c} \mathrm{vec}\left({\hat{G}}_{S}\left(f\right)\right)=\frac{1}{2}\left({\left(\Psi_{S}^{*}\left(f\right)\circ \Psi_{S}\left(f\right)\right)}^{†}\mathrm{vec}\left({\hat{C}}_{\varphi (f)}-\sigma_{v(f)}^{2}I\right)+{\left(\Psi_{S}^{*}\left(f^{\prime }\right)\circ \Psi_{S}\left(f^{\prime }\right)\right)}^{†}\mathrm{vec}\left({\hat{C}}_{\varphi (f^{\prime })}-\sigma_{v(f^{\prime })}^{2}I\right)\right). \end{array}$$

Observe that not all the gains can be calculated by means of the latter expression. In the case of odd $N_{c}$, f=0 has to be determined using (18), whereas f=0 and $f=N_{c}/2$ have to be computed via (18) for $N_{c}$ even.

The proposed method, labeled as  DR-W-MUSIC (Dictionary-Rotation Wideband MUSIC), is summarized in Algorithm 1.
**Algorithm 1** Dictionary-Rotation Wideband MUSIC1:$g\leftarrow {\left[\sigma_{1}^{2},\ldots ,\sigma_{G}^{2}\right]}^{T}$2:**for all**$f\in \{1,\ldots ,N_{c}\}$**do**3:$\;\;{\hat{C}}_{\varphi (f)}\leftarrow \frac{1}{K}\sum_{k=1}^{K}\varphi_{k}\left(f\right)\varphi_{k}^{H}\left(f\right)$4:$\;\;\bar{U}\left(f\right)\leftarrow$ compute noise basis (16)5:**end for**6:**for all**i∈{1,…,G}**do**7:$\;\;J_{i}\leftarrow 0$8:**for all**$\;\;f\in \{1,\ldots ,N_{c}\}$**do**9:$\;\;\;\;a\left(\theta_{i},f\right)\leftarrow \Gamma \left(\theta_{i},f\right)a\left(\theta_{i}\right)$ rotate dictionary10:$\;\;\;\;\psi_{i}\left(f\right)\leftarrow T\left(f\right)a\left(\theta_{i},f\right)$11:$\;\;\;\;J_{i}\leftarrow J_{i}+{∥\psi_{i}^{H}\left(f\right)\bar{U}\left(f\right)∥}_{2}^{-2}$12:**  end for**13:**end for**14:S←*L* larger values on  $J_{i}$15:**for all**$f\in \{1,\ldots ,N_{c}/2\}$**do**16:$\;\;\Psi_{S}\left(f\right)\leftarrow T\left(f\right)\left[a\left(\theta_{s_{1}},f\right),\ldots ,a\left(\theta_{s_{L}},f\right)\right]$17:$\;\;\Psi_{S}\left(f^{\prime }\right)\leftarrow T\left(f^{\prime }\right)\left[a\left(\theta_{s_{1}},f^{\prime }\right),\ldots ,a\left(\theta_{s_{L}},f^{\prime }\right)\right]$18:$\;\;\mathrm{vec}\left({\hat{G}}_{S}\left(f\right)\right),\mathrm{vec}\left({\hat{G}}_{S}\left(f^{\prime }\right)\right)\leftarrow$ compute using (20)19:**end for**

### 4.3. Dictionary-Rotation Wideband Spatial Smoothing

Note that Algorithm 1 requires $\mathrm{rank}({\hat{C}}_{\varphi }\left(f\right))>L$, i.e., the number of estimated paths (or angles) is bounded by the number of training periods and the number of observations, i.e., $L\leq \min\{T_{\mathrm{tr}},K\}$. Solutions to address rank deficient scenarios have been proposed in [41,42]. In particular, these solutions apply when $\mathrm{rank}({\hat{C}}_{\varphi }\left(f\right))<L$. Nevertheless, in the proposed scenario, it is desirable to reduce the training sequence length $T_{\mathrm{tr}}$ as much as possible. Unfortunately, we obtain a more involved setup for $\mathrm{rank}\left({\hat{C}}_{\varphi }\right)=T_{\mathrm{tr}}<L$. This scenario can be addressed by using spatial smoothing.

Spatial Smoothing (SS) is an array processing technique based on nested arrays with different antenna separation [43]. An effect similar to that of the nested arrays is obtained thanks to the use of sparse rulers [44]. Again, the proposed methods addressed the identification of signals lying in a single subspace, and we have to resort to dictionary rotations to exploit the wideband channel structure.

We start by introducing the sample covariance matrix vectorization $y\left(f\right)=\mathrm{vec}\left({\hat{C}}_{\varphi (f)}\right)$, i.e,

(21) $$\begin{array}{c} y\left(f\right)=\left(\Psi^{*}\left(f\right)\circ \Psi \left(f\right)\right)\mathrm{diag}\left(\tilde{G}\left(f\right)\right)+\sigma_{v(f)}^{2}e^{\prime }, \end{array}$$

where $e^{\prime }={\left[e_{1}^{T},\ldots ,e_{T_{\mathrm{tr}}}^{T}\right]}^{T}$. Observe that, due to the utilization of a perfect sparse ruler training T(f), $\left(\Psi^{*}\left(f\right)\circ \Psi \left(f\right)\right)$ contains the products corresponding to the spatial differences z∈{−M+1,M−1}. Conventional SS in [44] discards the repeated distances. However, this means employing 2M−1 elements out of $T_{\mathrm{tr}}^{2}$ from y(f) in (22). Hence, we propose to refine the estimation by averaging over all the samples from the same inter-antenna distance. To that end, let us introduce $y_{z}^{j}\left(f\right),j=1,\ldots ,J_{z},z=-M+1,\ldots ,M-1$ as the *j*-th sample of the *z*-th difference in y(f). Note that $J_{z}$ represents the number of samples available for the *z*-th difference, and its values depend on the sparse ruler structure and $T_{\mathrm{tr}}$. Next, we introduce the reduced vector $\overset{ˇ}{y}\left(f\right)\in C^{2M-1}$ with sorted elements ${\left[\overset{ˇ}{y}\left(f\right)\right]}_{z}=\frac{1}{J_{z}}\sum_{j=1}^{J_{z}}y_{z}^{j}\left(f\right)$ corresponding to the 2M−1 distances. Accordingly, we define the matrix Z(f) containing 2M−1 sorted rows from $\left(\Psi^{*}\left(f\right)\circ \Psi \left(f\right)\right)$. Thus, the reduced vector is

(22) $$\overset{ˇ}{y}\left(f\right)=Z\left(f\right)\mathrm{diag}\left(\tilde{G}\left(f\right)\right)+\sigma_{v(f)}^{2}e_{M}.$$

These differences in $\overset{ˇ}{y}\left(f\right)$ are next seen as a phase shift of the *M* differences to be estimated. Considering *M* overlapping subarrays, such that the *m*-th subarray comprises the differences {−M+m,m−1} in ${\overset{ˇ}{y}}_{m}\left(f\right)\in C^{M}$, the spatial smoothed matrix is obtained as follows: $\overset{ˇ}{Y}\left(f\right)=\frac{1}{M}\sum_{m=1}^{M}{\overset{ˇ}{y}}_{m}\left(f\right){\overset{ˇ}{y}}_{m}^{H}\left(f\right)$. As shown in [43], $\overset{ˇ}{Y}\left(f\right)={\overset{ˇ}{Y}}^{1/2}\left(f\right){\overset{ˇ}{Y}}^{1/2}\left(f\right)$ where the matrix ${\overset{ˇ}{Y}}^{1/2}\left(f\right)$ can be rewritten as

(23) $${\overset{ˇ}{Y}}^{1/2}\left(f\right)=\frac{1}{\sqrt{M}}\left(A\left(f\right)\tilde{G}\left(f\right)A^{H}\left(f\right)+\sigma_{v(f)}^{2}I_{M}\right).$$

Due to the beam squint effect, (23) has to be computed individually for each subcarrier *f*. Nevertheless, we desire to achieve the benefits of exploiting the structure of the wideband channel. In this regard, we employ $\sqrt{M}{\overset{ˇ}{Y}}^{1/2}\left(f\right)=U\left(f\right)\Lambda \left(f\right)U^{H}\left(f\right)$ as the sample covariance matrix in line 3 of Algorithm 1. Then, following Algorithm 1 steps, the *L* eigenvectors corresponding to the *L* largest eigenvalues are discarded to obtain an incomplete basis $\bar{U}\left(f\right)$ spanning the noise subspace. Hence, the wideband estimator incorporating the dictionary rotation capability of line 11 is updated as $J_{i}=J_{i}+{∥a^{H}\left(\theta_{i},f\right)\bar{U}\left(f\right)∥}_{2}^{-2}$, where $\theta_{i}$ is the *i*-th angle contained in the dictionary matrix A. To estimate the variance gains, we follow a similar procedure as that in Section 4.2 to derive the robust estimator

$$\begin{array}{cc} \mathrm{vec}({\hat{G}}_{S}\left(f\right)) & =\frac{1}{2}\left({\left(A_{S}^{*}\left(f\right)\circ A_{S}\left(f\right)\right)}^{†}\mathrm{vec}\left(\overset{˘}{Y}\left(f\right)\right)+(A_{S}^{*}\left(f^{\prime }\right)\circ A_{S}{\left(f^{\prime }\right)}^{†}\mathrm{vec}\left(\overset{˘}{Y}\left(f^{\prime }\right)\right)\right), \end{array}$$

with $A_{S}\left(f\right)=\left[a\left(\theta_{s_{1}},f\right),\ldots ,a\left(\theta_{s_{L}},f\right)\right]$ and $\overset{˘}{Y}\left(f\right)=\sqrt{M}{\overset{ˇ}{Y}}^{1/2}\left(f\right)-\sigma_{v(f)}^{2}I_{M}$. This procedure replaces lines 16 to 18, and completes the posed DR-W-SS method.

### 4.4. Dictionary Size

In this section, we provide some insight regarding the choice of the dictionary size *G*. Let us start by determining a sufficient condition for unique angular identification. To that end, we first define the Kruskal rank of a matrix X, krank(X). If krank(X)=x, then *x* is the maximum number such that any *x* columns of X are linearly independent.

**Theorem** **2.** *In the noiseless scenario, and assuming AoD lying on the dictionary A(f) and $\mathrm{rank}({\hat{C}}_{\varphi (f)})=L$, the inequality L<krank(Ψ(f)) guarantees the unique identification of the L AoD [41]. See [45] for the proof.*

**Figure 2:**
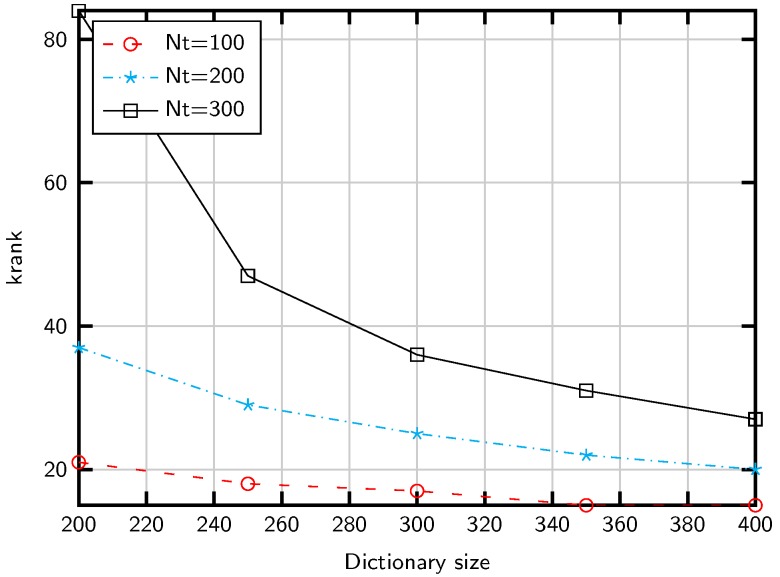
Kruskal rank upper bound for different array and dictionary sizes.

Therefore, when krank(A) is small, the probability of identifying false directions increases. To illustrate the dependence with *G*, we compute in Figure 2 an upper bound of the Kruskal rank for *G* and *M*. This upper bound is computationally feasible and takes into account only *z* consecutive columns, a reasonable approximationdue to the structure of A. As shown in the figure, large *G* values complicate the AoD detection, which suggests to reduce the dictionary size. However, this strategy entails a power leakagedue to basis mismatching, that is, a performance lossdue to off-grid AoDs. However, the basis mismatching is moderate in practical settingsdue to the finite angular resolution of the ULA. For instance, a typical inter-antenna spacing d=λ/2 entails an angular resolution of about $\frac{2}{M}$ radians [46], leading to $G\approx \frac{\Delta \vartheta \pi }{360º}M$ for an angular range Δϑ. That is, G≈M provides enough resolution to cover the sector Δϑ=120º. In addition, an alternative to increasing the dictionary size is to overestimate the number of channel propagation paths *L*. This strategy is explored in Section 6.2.

## 5. Delay-Gain Channel Estimator

As a means to estimate the channel, we propose a (DG) Delay-Gains estimator that exploits the structure of the wideband channels. This method employs the observations for all the subcarriers $\varphi_{k}\left(f\right)$ to estimate the frequency independent gains of each propagation path $g_{k}$ (cf. (6)). Thus, even in the case where certain frequencies present very poor SNR, their corresponding channels can be estimated by using the information from the remaining frequencies.

The covariance estimation procedure provides the measurement matrix $\Psi_{S}\left(f\right)$ and the estimated gain variances ${\hat{G}}_{S}\left(f\right)$ for all the frequencies. Using this information, we estimate the delay matrices B(f) and the gain vector $g_{k}$ from the observations gathered in the covariance estimation stage. Next, a robust low complexity estimator is calculated for the frequency selective channels.

First, notice that the observations in (8) can be rewritten as $\varphi_{k}\left(f\right)=\Psi_{S}\left(f\right)B_{S}\left(f\right)g_{S,k}+v_{k}\left(f\right)$, where $\Psi_{S}\left(f\right)$ was introduced in Section 4.2, $B_{S}\left(f\right)=\mathrm{diag}\left(\beta_{s_{1}}\left(f\right),\ldots ,\beta_{s_{L}}\left(f\right)\right)$ and $g_{S,k}$ contains the gains for the positions $s_{1},\ldots ,s_{L}$ of $g_{k}$. Then, we focus on the unknown vector $\zeta_{k}\left(f\right)=B_{S}\left(f\right)g_{S,k}$ that contains the channel gains affected by the shaping pulse. Accordingly, the linear Minimum Mean Squared Error (MMSE) estimator of $\zeta_{k}\left(f\right)$ reads as

(24) $$\begin{array}{cc} {\hat{\zeta }}_{k}\left(f\right)= & {\hat{G}}_{S}\left(f\right)\Psi_{S}^{H}\left(f\right){\left(\Psi_{S}\left(f\right){\hat{G}}_{S}\left(f\right)\Psi_{S}^{H}\left(f\right)+\sigma_{v(f)}^{2}I_{T_{\mathrm{tr}}}\right)}^{-1}\varphi_{k}\left(f\right). \end{array}$$

Once ${\hat{\zeta }}_{k}\left(f\right)$ is determined, we have to identify the matrices $B_{S}\left(f\right)$ before performing the estimation of $g_{S,k}$ jointly using the vectors ${\hat{\zeta }}_{k}\left(f\right)$ for all the frequencies. Since $B_{S}\left(f\right)$ contains samples of the shaping filter $p_{\mathrm{rc}}\left(\cdot \right)$ in the frequency domain, we obtain $B_{S}\left(f\right)$ by estimating the delays corresponding to the *L* channel paths. In particular, to determine delay for the *l*-th path $\tau_{l}$, we collect the entries as sociated with the *l*-th path for all the frequencies, i.e., ${\left[{\hat{\zeta }}_{k}\left(f\right)\right]}_{l}$ for $f=1,\ldots ,N_{c}$, in a vector. Then, we multiply this vector times the first *N* columns of the Discrete Fourier Transform (DFT) matrix, $F\in C^{N_{c}\times N}$, that is

(25) $$\begin{array}{cc} \zeta_{l,k} & =F^{H}{\left[\begin{array}{c} {\left[{\hat{\zeta }}_{k}\left(1\right)\right]}_{l},\ldots ,{\left[{\hat{\zeta }}_{k}\left(N_{c}\right)\right]}_{l} \end{array}\right]}^{T}. \end{array}$$

Observe now that $\zeta_{l,k}=g_{l,k}p_{\mathrm{rc}}\left(\tau_{l}\right)+w_{k}$ contains the aforementioned *N* stacked pulse samples $p_{\mathrm{rc}}\left(\tau_{l}\right)={\left[p_{\mathrm{rc}}\left(-\tau_{l}\right),p_{\mathrm{rc}}\left(T_{s}-\tau_{l}\right),\ldots ,p_{\mathrm{rc}}\left(\left(N-1\right)T_{s}-\tau_{l}\right)\right]}^{T}$, and the estimation noise $w_{k}∼N_{C}\left(0,\sigma_{w}^{2}I_{N_{c}}\right)$. Unfortunately, these samples are scaled by the unknown complex-valued channel gain $g_{l}$ of (2). Note that we assume that the error is statistically independent for different frequencies. Hence, for given shaping filter $p_{\mathrm{rc}}\left(t\right)$, we propose the following estimator function for τ

(26) $$\begin{array}{cc} D(\tau ) & =\sum_{k=1}^{K}\frac{|p_{\mathrm{rc}}^{T}\left(\tau \right)\zeta_{l,k}|}{∥p_{\mathrm{rc}}{\left(\tau \right)∥}_{2}{∥\zeta_{l,k}∥}_{2}}, \end{array}$$

where $p_{\mathrm{rc}}\left(\tau \right)={\left[p_{\mathrm{rc}}\left(-\tau \right),p_{\mathrm{rc}}\left(T_{s}-\tau \right),\ldots ,p_{\mathrm{rc}}\left(\left(N-1\right)T_{s}-\tau \right)\right]}^{T}$ contains the pulse samples for the delay τ. Unfortunately, D(τ) is a non-monotonic function. Thus, we minimize D(τ) over τ, e.g., by linear search over $\left[0,\left(N-1\right)T_{s}\right]$ to obtain the delay estimate ${\hat{\tau }}_{l}$. In the high SNR regime, it is enough to sound within the interval corresponding to the two samples of $p_{\mathrm{rc}}\left(\tau \right)$ with larger absolute values.

Recall that the channel gains are multiplied times $B_{S}\left(f\right)$ in the unknown vector $\zeta_{k}\left(f\right)$ of (24). Thus, employing the estimated delays ${\hat{\tau }}_{l}$, we compute ${\hat{B}}_{S}\left(f\right)$ for all frequencies (see (6)). To eventually estimate the channel gains $g_{S,k}$, we compute the minimum variance unbiased estimator [47] using information from all the frequencies, i.e., ${\hat{g}}_{S,k}={\hat{B}}^{†}{\hat{\zeta }}_{k}$, where $\hat{B}={\left[{\hat{B}}_{S}^{T}\left(1\right),\ldots ,{\hat{B}}_{S}^{T}\left(N_{c}\right)\right]}^{T}$ and ${\hat{\zeta }}_{k}={\left[{\hat{\zeta }}_{k}^{T}\left(1\right),\ldots ,{\hat{\zeta }}_{k}^{T}\left(N_{c}\right)\right]}^{T}$. Finally, by invoking (6), the estimated channel yields

(27) $${\hat{h}}_{k}\left(f\right)=A_{S}\left(f\right){\hat{B}}_{S}\left(f\right){\hat{g}}_{S,k}.$$

## 6. Simulation Results

The following setup is considered for the numerical experiments. We as sume a training sequence T generated from a Wichmann ruler of length $T_{\mathrm{tr}}$. The number of transmit antennas is M=200, while we consider a hybrid architecture using $N_{\mathrm{RF}}=2$ RF chains, $N_{c}=64$ subcarriers, and a dictionary of size G=400 with equally spaced angles within the range [0,π]. We consider the channel covariance model of (6) with *L* propagation paths uniformly distributed in [0,π]; different number of training periods *K*, and N=8 random delay taps uniformly distributed in $\left[0,N-1\right]T_{s}$, with $T_{s}=1/B$. The numerical results are averaged over 200 channel covariance realizations for independent users, and over the different subcarriers $N_{c}$. To evaluate the accuracy of the covariance identification, we employ the Normalized Mean Squared Error (NMSE) metric defined as $\mathrm{NMSE}=\frac{∥{\hat{C}}_{h}-C_{h}{∥}_{F}^{2}}{∥C_{h}{∥}_{F}^{2}}$ with the Frobenius norm ${∥\cdot ∥}_{F}$. In the case of channel estimation, we use the efficiency η∈[0,1] given by $\eta =\frac{|{\hat{h}}^{H}h|}{∥\hat{h}∥∥h∥}$, which is similar to the figures of merit in e.g., [26,34,48]. This insightful metric evaluates the signal power lost by beamforming using the estimated channels. Note that this metric is averaged over the number of channel blocks.

As a benchmark, we include an LS (known θ) curve that individually estimates the gain variances for each subcarrier using (18) as suming that the channel angles are known. That is, it does not exploit the symmetry with respect to the central frequency of (19) to estimate the variance gain.

### 6.1. Wideband Covariance Estimation

**Figure 3:**
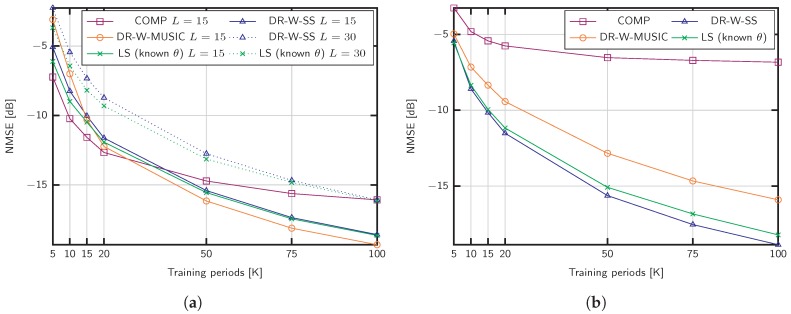
NMSE of different covariance estimation strategies for M=200 antennas: (**a**) SNR =15 dB, and L={15,30} propagation paths; (**b**) SNR =0 dB, and L=5 paths.

Figure 3a shows the NMSE obtained with the proposed algorithms. The training sequence length is set to $T_{\mathrm{tr}}=25$ and the channel parameters are adjusted according to massive MIMO settings with $f_{c}=5$ GHz, B=20 MHz, L={15,30} channel propagation paths, and a SNR of 15 dB. Using the same training sequence, we compare our methods with COMP in [34], which supports wideband estimation in the absence of beam squint. For L=15, the proposed methods achieve better performance than COMP when the number of training blocks *K* is large, but the accuracy of the estimation deteriorates when K<20. This behavior is motivated by the lack of similarity between the sample covariance matrix ${\hat{C}}_{\varphi (f)}$ and the actual channel covariance matrix $C_{\varphi (f)}$ that makes difficult the discrimination of the signal and noise subspaces (see Algorithm 1). Nevertheless, values of *K* above 50 are feasible in practical settings, since *K* is not restricted by the channel coherence time. We also include the case of L=30 channel paths in Figure 3a. Under such as sumption, COMP and DR-W-MUSIC do not applydue to the lack of angular sparsity, and the limited rank of the sample covariance matrices, respectively. Conversely, the DR-W-SS approach performs close to the benchmark with known angles, denoted by LS (known θ).

Figure 3a shows the NMSE for a mmWave setup with L=5, SNR =0 dB, a small signal bandwidth B=200 MHz and $f_{c}=28$ GHz. The robustness against the noise of the proposed strategies is remarkable, especially in the case of DR-W-SS. On the contrary, COMP does not achieve good performance results in this SNR regime compared to the proposed methods.

**Figure 4:**
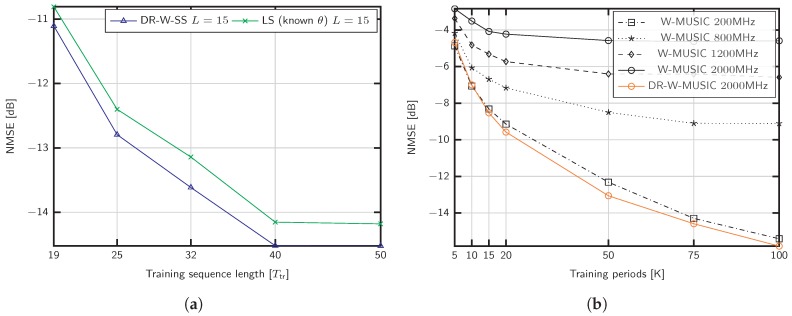
NMSE of covariance estimation for (**a**) M=100 antennas, L=50 propagation paths, K=50, SNR =15 dB, and different training sequence lengths; (**b**) M=200 antennas, L=5 propagation paths, and different signal bandwidths *B* for $f_{c}=28$ GHz.

To evaluate the performance impact of the training sequence length $T_{\mathrm{tr}}$, we fix the parameters to M=100, L=50, SNR =15 dB and K=50 in Figure 4a. The training sequence is shorter than the number of paths and takes the values 19, 25, 32, 40, 50, to show the capability of DR-W-SS in the challenging scenario $L>T_{\mathrm{tr}}$. As shown, the proposed method performs better than the LS benchmark with known support S.

We now evaluate the consequences of neglecting the beam squint effect for the setup considered in Figure 3a, that is, $f_{c}=28$ GHz and different signal bandwidths, B=200,800,1200,2000 MHz. The methods referred to as W-MUSIC consist of Algorithm 1 ignoring the dictionary rotation. Figure 4b exhibits the performance impact of beam squintdue to the lack of a common subspace for different frequencies. As shown, the proposed dictionary rotation is able to exploit the structure even in the case of B=2000 MHz.

### 6.2. Covariance Subspace Estimation

In the previous numerical examples, the number of paths *L* was as sumed to be known. Subspace estimation methods, like the one in [49], can be used to estimate *L* by comparing the differences between consecutive eigenvalues of the sample covariance matrix with a threshold ϵ. In the wideband case, we employ the information of all frequencies jointly, to increase the detection accuracy. Moreover, the computational cost over DR-W-MUSIC and DR-W-SS is negligible while it is significant for other approaches not performing a subspace discrimination.

**Figure 5:**
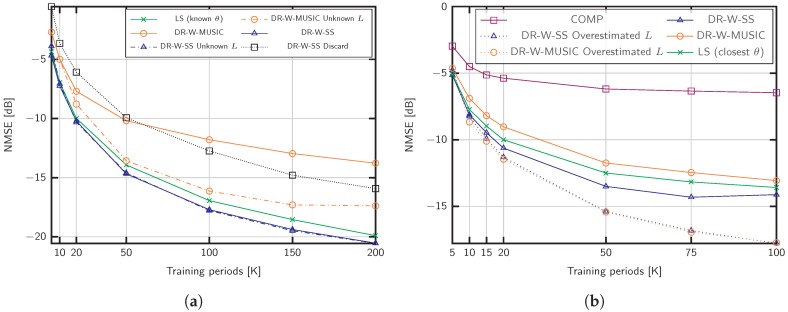
NMSE of covariance estimation for (**a**) M=200 antennas, L=15 propagation paths, $T_{\mathrm{tr}}=50$, SNR = 0 dB, and subspace estimation; (**b**) M=200, SNR = 0 dB, L=5 propagation paths, and off-grid AoDs.

Figure 5a shows the robustness against uncertainty in the number of channel propagation paths *L*. We employ Algorithm 2 in [49] and empirically adjust the parameter ϵ for the different estimation methods. For the considered scenario with SNR = 0 dB, we set the threshold as ϵ=0.25 for DR-W-MUSIC and ϵ=0.01 for DR-W-SS. Remarkably, the performance obtained with subspace estimation for DR-W-SS is similar to that of the strategies with known *L*. In particular, for DR-W-MUSIC, overestimating the number of channel paths *L* improves the performance. This observation reveals that the support identified by Algorithm 1 with known *L* was incorrect. Recall that the support S comprises the angles corresponding to the *L* larger values on the estimator function $J_{i}$. Under the strong noise conditions considered in Figure 5a, some angles belonging to the covariance support might be ignored (see line 14 of Algorithm 1). Instead, step 14 selects angles in the dictionary whose steering vectors are linearly dependent. Thus, overestimating *L* enables a better detection of the channel covariance subspace where angles with lower $J_{i}$ are included in S. In addition, we compare the effect of discarding the repeated distances in DR-W-SS, as performed in conventional SS [44]. In Figure 5a, this strategy is labeled DR-W-SS Discard. As shown, for $T_{\mathrm{tr}}=50$, the performance loss is significant.

In Section 4.4, we discussed the advantages and practical orientation of moderate dictionary sizes. Nevertheless, we investigate the effect of off-grid AoDs in Figure 5b for the mmWave setup and B=200 MHz. The error floordue to basis mismatch is clear. As an alternative to increase *G*, we propose to compensate the basis inaccuracy by overestimating the number of channel paths *L*. This way, part of the power leakage is recovered with the additional AoDs. These approaches are denoted DR-W-MUSIC Overestimated *L* and DR-W-SS Overestimated *L*. For the LS estimator with known angles, we choose the closest dictionary angles to the real ones.

### 6.3. Wideband Channel Estimation

**Figure 6:**
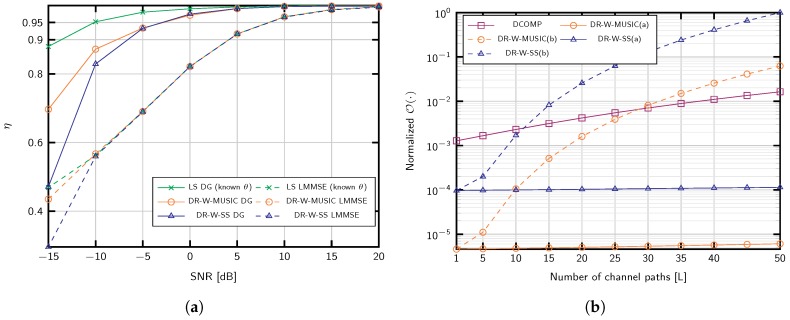
(**a**) efficiency of channel estimation for M=200 antennas, L=5 propagation paths, $T_{\mathrm{tr}}=25$, and K=100 training periods; (**b**) normalized computational complexity for M=200 antennas, dictionary size G=2M, K=150 training periods, training length $T_{\mathrm{tr}}=50$, $N_{c}=64$ subcarriers, and different number of paths.

Figure 6a shows the performance of the channel estimator with the covariance identified and the two different channel estimation methods: LMMSE [21,25,47]

(28) $${\hat{h}}_{k}\left(f\right)={\hat{C}}_{h(f)}T^{H}{\left(T{\hat{C}}_{h(f)}T^{H}+\sigma_{v(f)}^{2}I_{T_{\mathrm{tr}}}\right)}^{-1}\varphi_{k}\left(f\right)$$

and the proposed DG estimator of (27). The number of channel blocks is set to K=100, and the number of delay candidates τ considered in (26) is 20 *N*. Since the proposed DG method uses the information of all the subcarriers to produce the estimates, it is very robust against noise and results in a considerable performance gain. Indeed, more than 80% of the signal power is captured at −10 dB with the proposed strategies.

### 6.4. Computational Complexity

Table 1 summarizes the computational complexity of the proposed methods. Recall that a fast covariance estimation is important, but not as critical as the actual channel estimation. Indeed, we only employ a training stage contrarily to other approaches like [21,37].

For the favorable scenario, DR-W-MUSIC(a) and DR-W-SS(a), the gain variances are estimated using well-conditioned measurement matrices, while (18) with the Khatri–Rao product is considered for MUSIC(b) and DR-W-SS(b). The complexities have been calculated as suming $T_{\mathrm{tr}}>L$ for DR-W-MUSIC and COMP. It is noticeable that neither DR-W-MUSIC nor COMP depend on the number of antennas, although both of them linearly depend on the dictionary size *G*. The number of channel taps *N* is also relatively small, and *T* is the number of τ candidates evaluated to estimate the delays using (26). Since COMP and DR-W-MUSIC apply to scenarios where *L* and $T_{\mathrm{tr}}$ are very small compared to *M* or *G*, the complexity of the latter is smaller in general. Furthermore, the number of steps performed by COMP is larger, and its computational burden depends on the number of training periods *K*.

When L<≮M, the computational complexity virtually depends on the number of antennas. Hence, DR-W-SS and DR-W-MUSIC establish a trade-off among $T_{\mathrm{tr}}$, *L* and *M*, since we could use the less complex option by adjusting $T_{\mathrm{tr}}$ and *K*. The effect of increasing *L* is depicted in Figure 6b. Interestingly, in the region where the channel is sparse and Dynamic COMP (DCOMP) applies, L<10, the computational complexity of the proposed approaches is lower than that of DCOMP.

We now differentiate between the operations computed once using the estimated covariance matrix, referred to as “Computing Estimator”, and the channel estimation for each observation, referred to as “Channel Estimation”. Remarkably, the complexity of computing the proposed estimator (DG) linearly depends on *M*, cf. (24)–(26) whereas for the LMMSE estimator this dependency is quadratic.

Regarding the actual channel estimation, in scenarios suitable for DR-W-MUSIC with $L<T_{\mathrm{tr}}$, DG channel estimation is more efficient. This applies to mmWave frequencies and other massive MIMO scenarios, e.g., [8,37,50,51,52,53]. Conversely, LMMSE estimator might be less complex for large number of channel propagation paths, i.e., $L>\sqrt{M}$.

## 7. Conclusions

In this work, we proposed a covariance and channel estimation method for FDD wideband hybrid massive MIMO systems. The numerical experiments showed the good performance of the posed covariance estimation approaches compared to other methods in the literature, even when considering very short training sequence lengths. In addition, we developed a robust channel estimator with computational complexity depending linearly on the number of antennas. Both facts contributed to a reduction in the channel training overhead. Finally, we evaluated the performance of the proposed scheme for a wide variety of scenarios. The extension to Uniform Planar Array (UPA) antenna arrangements, and the use of shorter training sequences is an interesting research line for future works.

## Figures and Tables

**Table 1:** Complexitiesof estimation methods.

	Covariance Estimation
DR-W-MUSIC(a)	$O\left(N_{c}\left(T_{\mathrm{tr}}^{3}+\left(T_{\mathrm{tr}}^{2}+T_{\mathrm{tr}}\right)G+L^{2}T_{\mathrm{tr}}+2LT_{\mathrm{tr}}^{2}\right)\right)$
DR-W-MUSIC(b)	$O\left(N_{c}\left(T_{\mathrm{tr}}^{3}+\left(T_{\mathrm{tr}}^{2}+T_{\mathrm{tr}}\right)G+L^{4}T_{\mathrm{tr}}^{2}+L^{2}T_{\mathrm{tr}}^{2}\right)\right)$
DCOMP	$O(N_{c}\left(2KT_{\mathrm{tr}}^{2}G+KL^{3}T_{\mathrm{tr}}+2KT_{\mathrm{tr}}^{2}\left(L^{2}+L\right)+KLT_{\mathrm{tr}}^{3}\right))$
DR-W-SS(a)	$O\left(N_{c}\left(M^{2}+M^{3}+\left(M^{2}+M\right)G+L^{2}M+2LM^{2}\right)\right)$
DR-W-SS(b)	$O\left(N_{c}\left(M^{2}+M^{3}+\left(M^{2}+M\right)G+L^{4}M^{2}+L^{2}M^{2}\right)\right)$
	**Computing Estimator**
DG	$O\left(N_{c}\left(3L^{2}T_{\mathrm{tr}}+T_{\mathrm{tr}}^{3}+NL+L^{3}+ML^{2}+ML\right)+KNTL\right)$
LMMSE (28)	$O(N_{c}\left(3M^{2}T_{\mathrm{tr}}+T_{\mathrm{tr}}^{3}\right))$
	**Channel Estimation**
DG	$O\left(N_{c}\left(LT_{\mathrm{tr}}+ML+L^{2}\right)\right)$
LMMSE (28)	$O(N_{c}MT_{\mathrm{tr}})$

## Appendix A

To prove the linear independence, we consider two cases: (1) If the columns of $\Psi_{S}\left(f\right)$ are linearly independent, the same property holds for the columns of $\Psi_{S}^{*}\left(f\right)\circ \Psi_{S}\left(f\right)$. (2) Consider that the *i*-th column of $\Psi_{S}\left(f\right)$, $\psi_{S_{i}}$ for notation simplicity, is a linear combination of the columns whose indices are in the set T⊆S, i.e., $\psi_{S_{i}}=\sum_{j=1}^{\left|T\right|}c_{j}\psi_{T_{j}}$ with $\psi_{T_{j}}\neq 0,\forall j\in T$. Then, for the *i*-th column of $\left(\Psi_{S}^{*}\left(f\right)\circ \Psi_{S}\left(f\right)\right)$, we obtain

(A1) $$\sum_{j=1}^{\left|T\right|}c_{j}^{*}\psi_{T_{j}}^{*}⊗\sum_{n=1}^{\left|T\right|}c_{n}\psi_{T_{n}}=\sum_{j=1}^{\left|T\right|}\sum_{n=1}^{\left|T\right|}c_{j}^{*}\psi_{T_{j}}^{*}⊗c_{n}\psi_{T_{n}}.$$

Notice that it is not possible to write this column as a linear combination of the form $\sum_{j=1}^{\left|T\right|}b_{j}\psi_{T_{j}}^{*}⊗\psi_{T_{j}}$ unless $\psi_{T_{j}}=\sum_{n=1}^{\left|T\right|}c_{n}\psi_{T_{n}},\;\forall j$. This condition holds only if $\psi_{S_{i}}=c_{j}\psi_{T_{j}}$.
